# Formation processes, size changes, and properties of nanosheets derived from exfoliation of soft layered inorganic–organic composites†

**DOI:** 10.1039/d0na00084a

**Published:** 2020-01-30

**Authors:** Ryosuke Mizuguchi, Hiroaki Imai, Yuya Oaki

**Affiliations:** Department of Applied Chemistry, Faculty of Science and Technology, Keio University 3-14-1 Hiyoshi, Kohoku-ku Yokohama 223-8522 Japan oakiyuya@applc.keio.ac.jp; JST, PRESTO 4-1-8 Honcho Kawaguchi Saitama 332-0012 Japan

## Abstract

Exfoliation is a general route to obtain two-dimensional (2D) nanomaterials. A variety of methods have been developed for controlled exfoliation of layered materials based on stacking *via* van der Waals interaction, such as graphite and transition-metal dichalcogenides. On the other hand, rigid layered materials consisting of inorganic layers and interlayer metal ions stacked *via* electrostatic interaction, such as transition-metal oxides and clays, have a limited number of exfoliation methods. Here we studied a new exfoliation route through formation of soft layered composites. Intercalation of guest organic molecules changed rigid inorganic layered compounds into soft layered composites with stacking *via* van der Waals interaction. The soft layered inorganic–organic composites were exfoliated into surface-modified nanosheets in organic media. The layered composites showed swelling with dispersion in organic media. The time-course analyses suggest that the layered composites were simultaneously exfoliated in the vertical direction and fractured in the lateral direction. Thinner and smaller nanosheets were obtained with an increase in the exfoliation time. Although the resultant nanosheets gradually aggregated in the colloidal liquid, the original dispersion state was recovered with sonication for 5 min at room temperature. This exfoliation route using soft layered composites can be used in the synthesis and application of a variety of 2D nanomaterials.

## Abstract 2

A new exfoliation route is developed to obtain 2D nanomaterials through formation of soft layered inorganic–organic composites.

## Graphical abstract

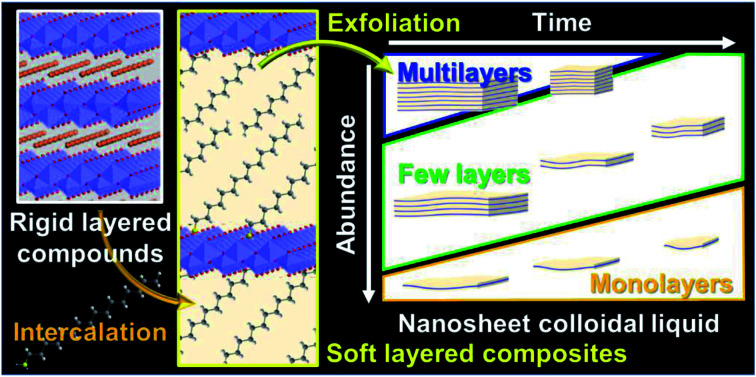

## Introduction

**Fig. 1:**
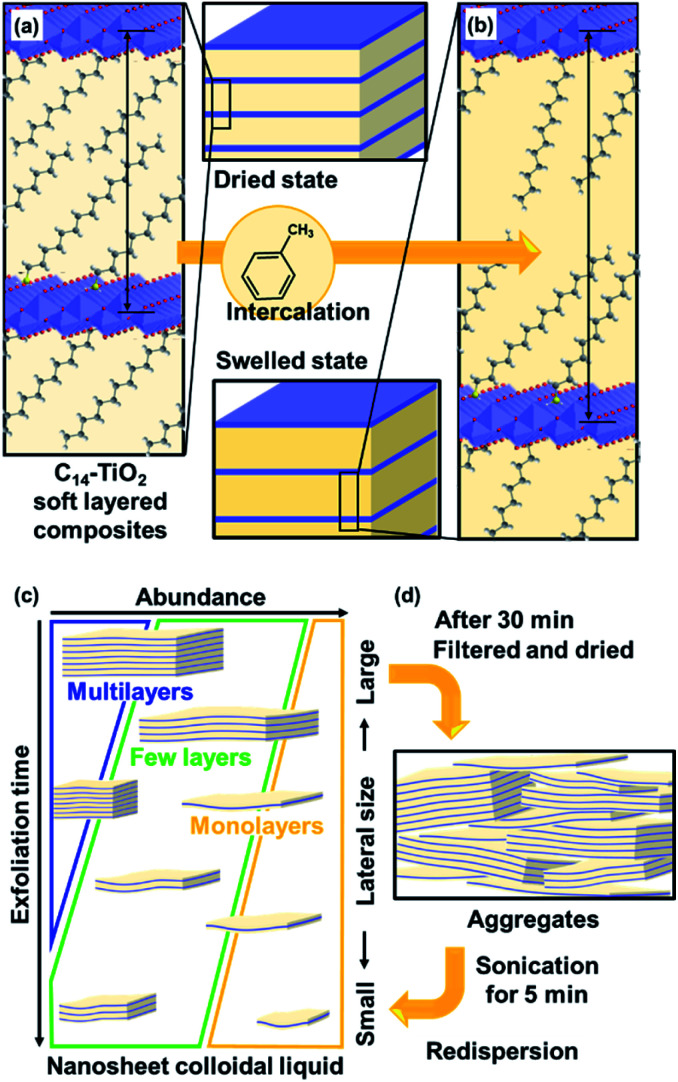
Exfoliation of soft layered organic–inorganic composites into surface-modified nanosheets. (a) Layered C_14_–TiO_2_ with intercalation of C_14_–NH_2_ in the interlayer space. (b) Swelling of the layered C_14_–TiO_2_ with intercalation of toluene in the hydrophobic interlayer space. (c) Size-change behavior of the C_14_–TiO_2_ nanosheets with an increase in the exfoliation time. (d) Aggregation and redispersion behavior of the C_14_–TiO_2_ nanosheets.

Two-dimensional (2D) nanomaterials have attracted much interest in recent years.^1–10^ A variety of layered materials are exfoliated into nanosheets, such as monolayer and few-layer nanosheets.^11–19^ Anisotropic nanostructures exhibit characteristic properties different from those of bulk materials and isotropic morphologies.^20–28^ Exfoliation is a general route to obtain nanosheets from precursor layered materials. However, exfoliation routes face a number of challenges, such as control of the size, improvement of the yield, and modification of the nanosheet surface. Development of new exfoliation methods is required to achieve controlled synthesis of 2D nanomaterials and their practical applications. Here we studied a recent new exfoliation method through formation of soft layered composites. The precursor layered composites consisting of inorganic layers and organic interlayer guests are exfoliated into surface-modified nanosheets in organic dispersion media (Fig. 1). This exfoliation method has potential for synthesis of nanosheets with controlled yield, size, and surface chemistry.^29–37^ The exfoliation behavior can be changed with different combinations of interlayer guests and dispersion media. However, the exfoliation processes and stability of the resultant nanosheets have not been fully studied in previous work. Elucidation of the processes is important for application of the exfoliation method to design a variety of 2D nanomaterials.

Exfoliation methods differ for different structures of layered compounds. Layered structures, such as transition-metal dichalcogenides, graphite, black phosphor, and covalent organic frameworks, are formed by stacking *via* van der Waals interaction. A variety of exfoliation methods have been studied for these layered materials, such as mechanical and sonication methods.^1–5,11,16,17^ Layered compounds based on stacking *via* electrostatic interaction, such as clay, layered double hydroxides, and transition-metal oxides, have charged host layers and guest interlayer ions. This type of layered structure is regarded as more rigid than that based on van der Waals interaction. In general, exfoliation of these rigid layered compounds is initiated by intercalation of bulky ions in the interlayer space.^12,29,30,38,39^ The subsequent swelling with aqueous media facilitates exfoliation into the nanosheets against the electrostatic interaction between the host layers and guest ions. However, the exfoliation behavior is not fully controlled by factors related to the structure and experimental conditions for synthesis of nanosheets with a controlled size and surface in high yield. In recent years, layered composites and hybrids consisting of inorganic layers and interlayer organic guests have been exfoliated into nanosheets.^40–48^ These precursor layered structures are regarded as the soft type based on van der Waals interaction of the interlayer guest molecules. Our group has found that the surface, size, and yield of transition-metal-oxide nanosheets are controlled by guest-medium combinations through exfoliation of the precursor layered composites (Fig. 1).^29,30,34,35^ However, the exfoliation processes were not studied in our previous work. Mechanistic studies including the processes and related parameters were reported for other exfoliation methods.^49–52^ Therefore, here we studied the exfoliation processes and stability of the resultant nanosheets to achieve controlled syntheses using layered composites.

After exfoliation, the dispersion stability of the nanosheets is significant for their practical use. In general, the nanosheets are obtained in a colloidal dispersion liquid. The charged nanosheets derived from the rigid layered structures undergo restacking and aggregation with counter ions *via* electrostatic interaction. On the other hand, the surface-modified nanosheets derived from the layered composites are restacked *via* van der Waals interaction. Therefore, the original dispersion state can recover more easily with a simple treatment, such as sonication. The present work focused on the dispersion stability of the surface-modified nanosheets (Fig. 1d). Furthermore, the methods and conditions for reproducing the original dispersion state were studied for practical use of the surface-modified nanosheets. Our intention here is to elucidate the exfoliation processes of the soft layered composites and stability of the nanosheets.

## Results and discussion

### Formation of soft layered composites

**Fig. 2:**
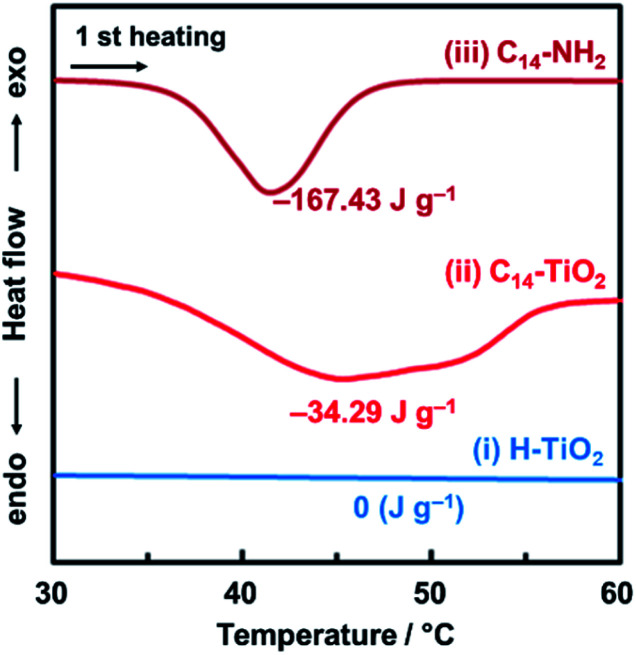
DSC thermograms of the H–TiO_2_ (i), layered C_14_–TiO_2_ (ii), and C_14_–NH_2_ (iii) at the first heating under a nitrogen atmosphere.

The original inorganic layered compounds are comprised of charged layers and interlayer counter ions stacked *via* electrostatic interaction. Layered inorganic–organic composites have interlayer guest molecules stacked *via* van der Waals interaction. In general, van der Waals interaction is weaker than the electrostatic one. The layered composites contain domains consisting of weaker interlayer interaction compared with the original one. The layered C_14_–TiO_2_ showed an endothermic peak around 40 °C with heating in the chart of differential scanning calorimetry (DSC) (Fig. 2). The endothermic peak originating from molecular motion near the melting point was observed at around 40 °C for pure C_14_–NH_2_, whereas the layered titanate with intercalation of protons (H–TiO_2_) has no peak in this temperature range (Fig. 2). These results imply that the layered C_14_–TiO_2_ shows thermal motion of the interlayer C_14_–NH_2_ at around 40 °C. The thermal motion can easily initiate the exfoliation into the surface-modified nanosheets in organic media. Therefore, the layered composites are regarded as the soft type compared with the pristine compound.

### Initial stage for exfoliation of the layered composite in organic media

**Fig. 3:**
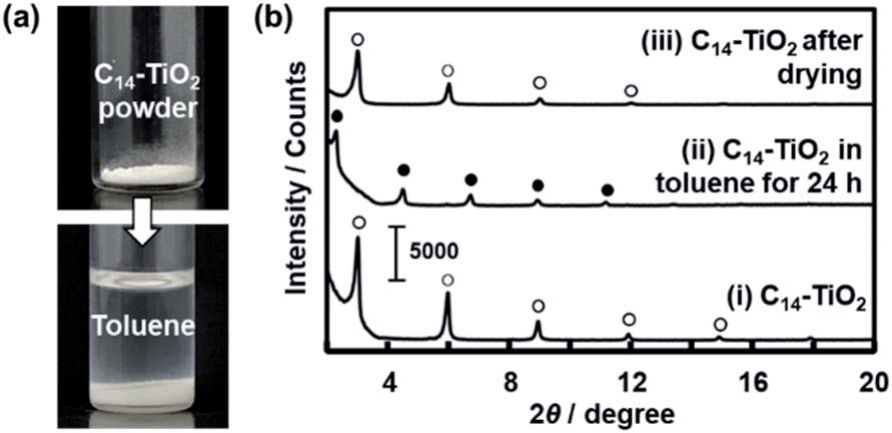
Swelling behavior of the layered C_14_–TiO_2_ with immersion in toluene. (a) Photographs of the C_14_–TiO_2_ powder before (the upper panel) and after (the lower panel) addition of toluene. (b) XRD patterns of the layered C_14_–TiO_2_ powder in the original dried state (i), the slurry with immersion in toluene (ii), and the dried state after evaporation of toluene (iii). The open and filled circles represent the peaks corresponding to the original layered C_14_–TiO_2_ and its swollen state with toluene, respectively.

We studied the initial stage of the exfoliation process to find whether the layered composite is swollen or not. The precursor layered composite was prepared by intercalation of an organic guest in a transition-metal oxide according to our previous report.^29^ Tetradecylamine (C_14_H_29_NH_2_, C_14_–NH_2_) was intercalated into layered titanate. The detailed procedure is described in the ESI.† The layered composite of titanate and C_14_–NH_2_ (C_14_–TiO_2_) was measured in toluene at 60 °C (Fig. 3). The powder of the layered C_14_–TiO_2_ showed a slight increase in volume in the photograph after immersion in toluene for 120 h (Fig. 3a).

The changes of the interlayer distance and crystallinity were studied by X-ray diffraction (XRD) (Fig. 3b). A sample holder was filled with about 10 mg of C_14_–TiO_2_ dried powder for XRD measurement. After the measurement, about 2 cm^3^ of toluene was poured in the sample holder. The sample holder was sealed and maintained at 60 °C for 24 h. Then, the XRD patterns were measured in the wet and subsequent dried states (patterns (ii) and (iii) in Fig. 3b). The precursor layered C_14_–TiO_2_ showed peaks at 2*θ* = 2.99°, 5.97°, 8.92°, and 11.3° corresponding to the lattice spacing *d*_0_/*n* (*n* = 1, 2, 3, 4) on the assumption that the interlayer distance *d*_0_ = 2.92 nm, respectively (pattern (i) with open circles in Fig. 3b). The interlayer distance shifted to *d*_0_ = 3.82 nm after immersion in toluene (pattern (ii) with the filled circles in Fig. 3b) and then recovered to *d*_0_ = 2.95 nm after evaporation of toluene (pattern (iii) with the open circles in Fig. 3b). The peak shift and recovery were reversibly observed for the wet and dried states of the layered C_14_–TiO_2_ (Fig. S1 in the ESI†). The peak shift was observed within 10 min after immersion in toluene (Fig. S1 in the ESI†). In Fig. 3b, the full-width at half-maximum of the *d*_0_ peak was 0.116 degree for the precursor layered C_14_–TiO_2_ powder, 0.111 degree after immersion in toluene, and 0.239 degree after drying. The peak broadening indicates lowering of the crystallinity after the drying.

These results suggest that the layered C_14_–TiO_2_ is swollen with introduction of toluene in the hydrophobic interlayer space (Fig. 1a and b). While the crystallinity of the layered structure was preserved with swelling, the dried state showed a lower crystallinity. The interlayer alkyl chains have a stable all-*trans* conformation in the original layered C_14_–TiO_2_ and in its swollen state with toluene. On the other hand, the conformation is distorted after evaporation of toluene. This swelling behavior initiates the exfoliation of the layered C_14_–TiO_2_ into nanosheets.

### Changes in the lateral size, thickness and yield

**Fig. 4:**
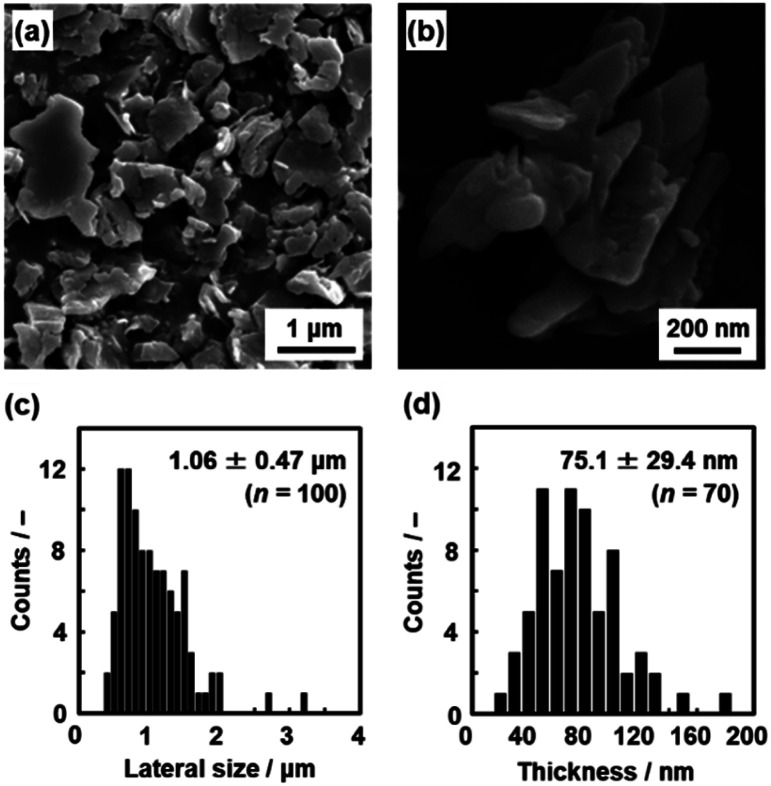
Size of the precursor layered C_14_–TiO_2_. (a and b) SEM images. (c and d) Histograms of the lateral size (c) and thickness (d) with the average size, standard deviation, and sample number (*n*).

**Fig. 5:**
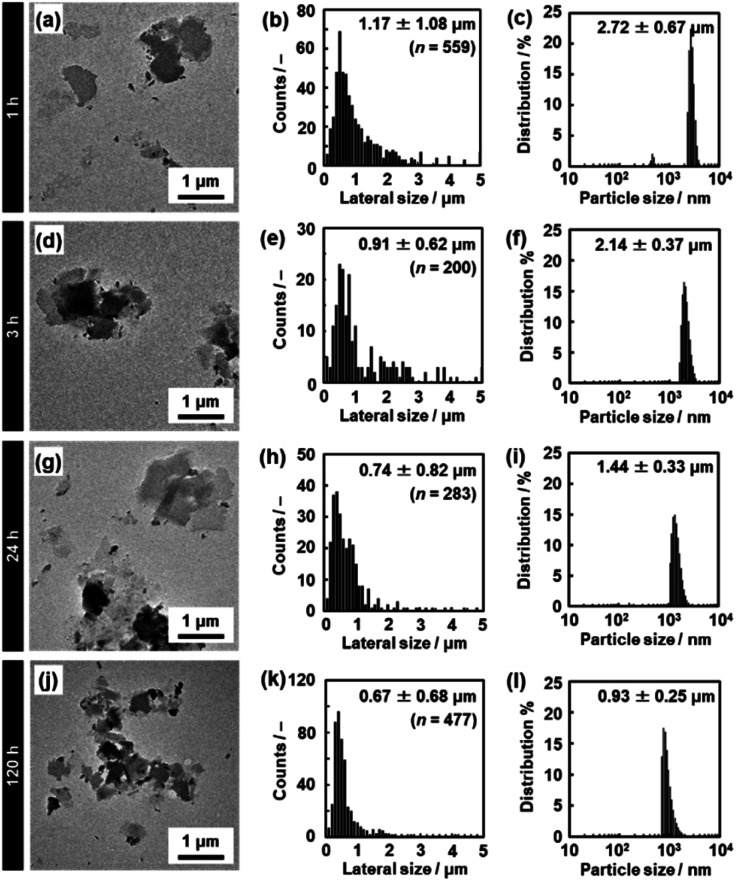
TEM images (a, d, g and j), histograms of the lateral size (b, e, h and k), and DLS charts (c, f, i and l) of the C_14_–TiO_2_ nanosheets after exfoliation for 1 h (a–c), 3 h (d–f), 24 h (g–i), and 120 h (j–l) at 60 °C. The data for 5 h and 72 h are listed in Fig. S2 in the ESI.†

**Fig. 6:**
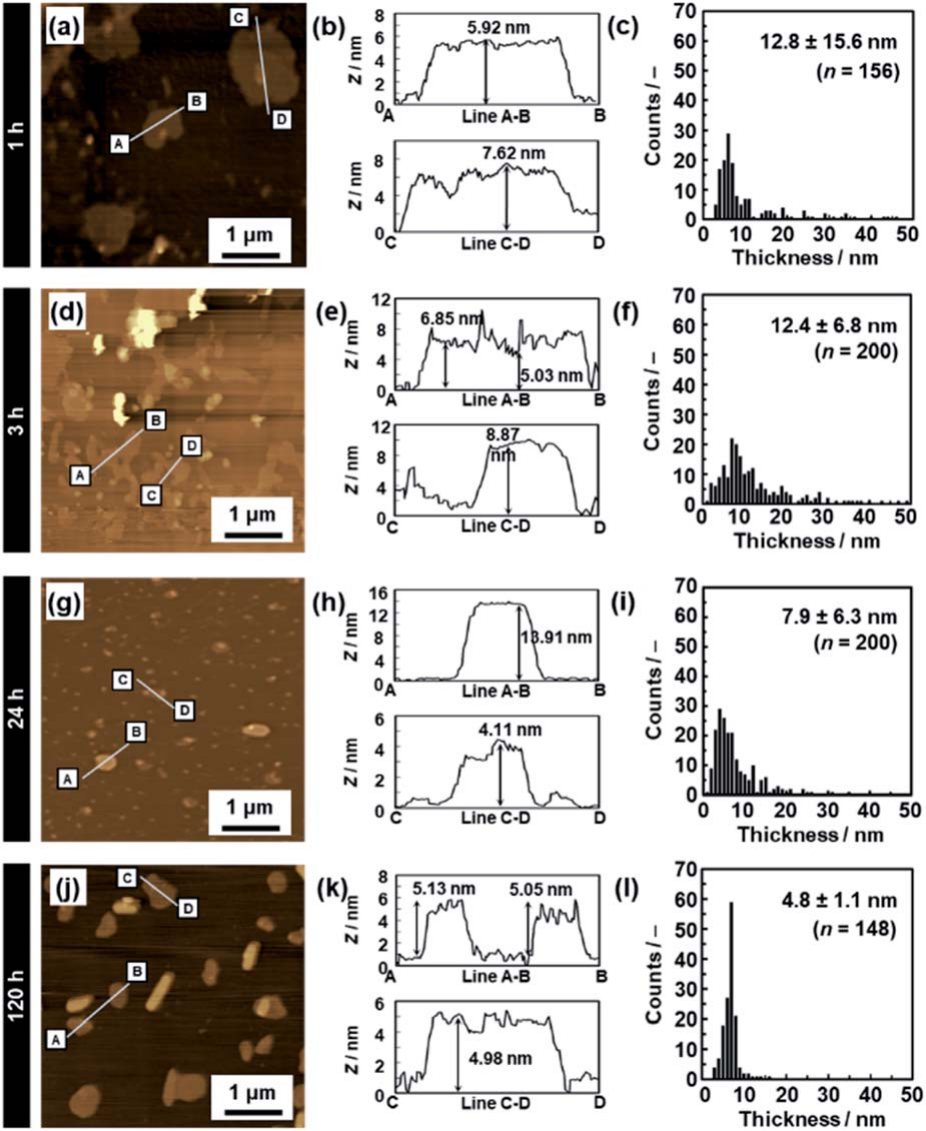
AFM images (a, d, g and j), their height profiles (b, e, h and k), and histograms of the height (c, f, i and l) of the C_14_–TiO_2_ nanosheets after exfoliation for 1 h (a–c), 3 h (d–f), 24 h (g–i), and 120 h (j–l). The data for 5 h and 72 h are listed in Fig. S2 in the ESI.†

**Fig. 7:**
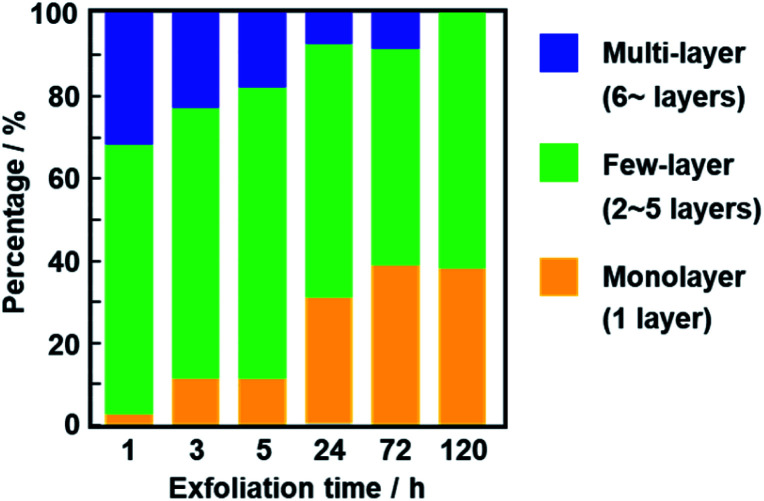
Percentage of monolayer (*N* = 1), few-layer (*N* = 2–5), and multi-layer (*N* > 5) nanosheets after exfoliation for 1, 3, 5, 24, 72, and 120 h.

**Fig. 8:**
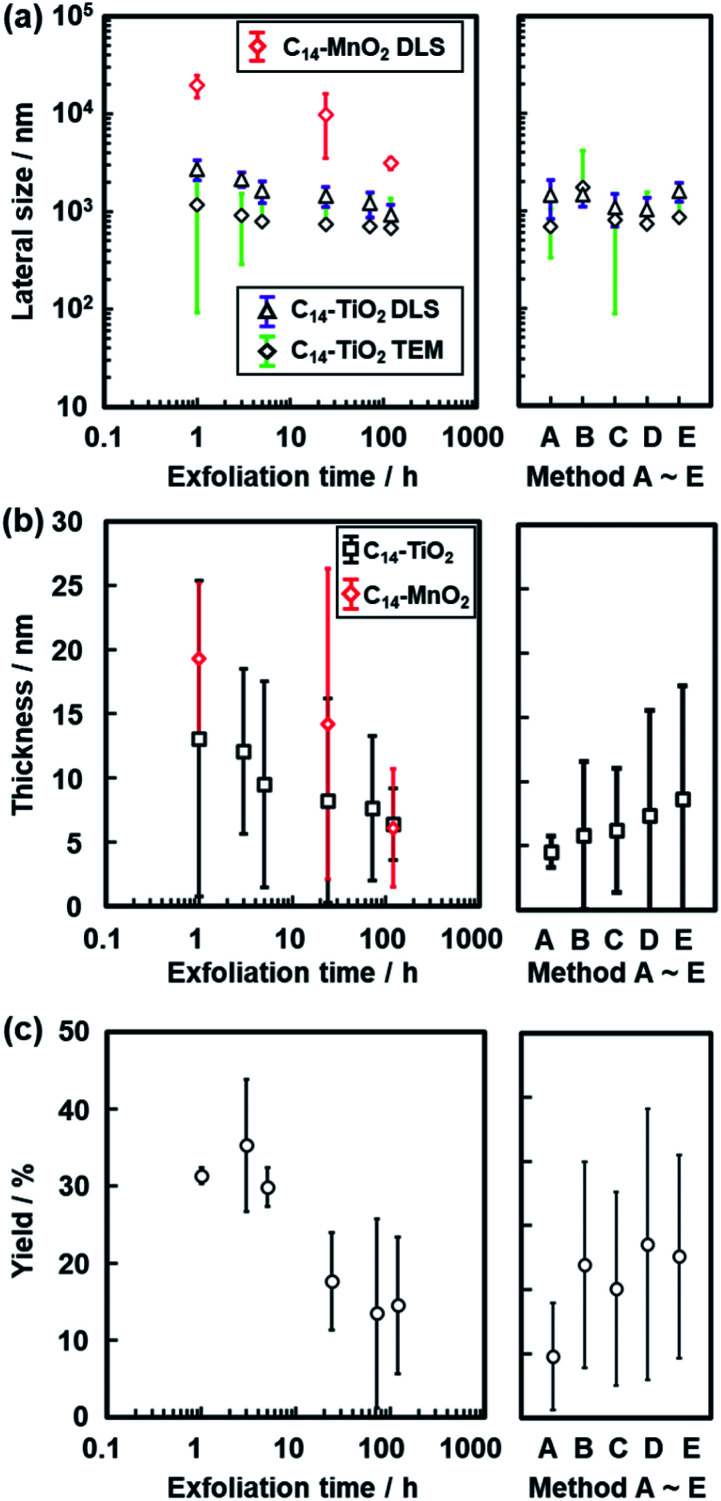
Changes in the size of the C_14_–TiO_2_ and C_14_–MnO_2_ nanosheets with an increase in the exfoliation time. (a) Changes in the lateral size measured by DLS (triangles) and TEM (diamonds). (b) Changes in the thickness measured by AFM. (c) Changes in the yield. The data in the right panels were obtained by reference exfoliation methods A–E for the layered C_14_–TiO_2_.

The layered C_14_–TiO_2_ was dispersed in toluene at 60 °C under stirring for 1 to 120 h. The resultant dispersion liquid was filtered using a membrane filter with 2.0 μm pore size to remove the unexfoliated materials and aggregated nanosheets because the precursor layered C_14_–TiO_2_ exhibited a plate shape with 1.06 ± 0.47 μm lateral size and 75.1 ± 29.4 nm thickness in the scanning electron microscopy (SEM) images (Fig. 4). After filtration, the dispersion liquid was used to analyze the lateral size and thickness of the surface-modified C_14_–TiO_2_ nanosheets by transmission electron microscopy (TEM), dynamic light scattering (DLS) analysis, and atomic force microscopy (AFM) (Fig. 5–8).

The lateral size of the nanosheets was estimated to be 1.17 ± 1.08 μm from the TEM images and 2.72 ± 0.67 μm from DLS analysis after 1 h of exfoliation (Fig. 5a–c). The lateral size decreased with an increase in the exfoliation time (Fig. 5d–l, 8a and S2 in the ESI†). The sizes estimated from the TEM image and DLS analysis are correlated with each other, even though these sizes are not the same values (Fig. 8a). The correlation of the sizes between the TEM image and DLS analysis was reported in previous studies.^35,53^ These results suggest that DLS analysis can be used not for accurate characterization but for rough estimation of the lateral size instead of the time-consuming microscopy observations.

The average thickness of the nanosheets was 12.8 ± 15.6 nm after 1 h in the AFM images (Fig. 6a–c). Thinner nanosheets were obtained with an increase in the exfoliation time from 1 h to 120 h (Fig. 6d–l, 8b and S2 in the ESI†). The composition, namely the amount of surface molecules, was not changed for the C_14_–TiO_2_ nanosheets even after dispersion in toluene for 120 h (Fig. S3 in the ESI†). The distribution of the thickness is ascribed to the mixture of mono-, few-, and multi-layered nanosheets in the dispersion liquid (Fig. 5 and 6). According to our previous reports, both the sides of the resultant nanosheets were modified by alkylamine.^29^ The thickness of the monolayered C_14_–TiO_2_ was around 2.0 nm as obtained by AFM analysis in our previous report,^29^ whereas the bare titanate monolayer showed a thickness of around 0.7 nm.^12^ The interlayer distance of the layered C_14_–TiO_2_ was estimated to be 2.92 nm by XRD analysis (Fig. 3b). Here the range of thickness is 1.50–4.50 nm for the monolayer (layer number *N* = 1), 4.50–16.5 nm for the few-layer (*N* = 2–5), and thicker than 16.5 nm for the multi-layer (*N* > 5) nanosheets on the basis of these structural models (Fig. S4 in the ESI†). The percentage of monolayer, few-layer, and multi-layer nanosheets gradually varied with the exfoliation time (Fig. 7). The number of thinner nanosheets, such as monolayer and few-layer nanosheets, increased with an increase in the exfoliation time, whereas that of the thicker multilayer decreased. These observations imply that the layered composites are exfoliated into nanosheets with fragmentation in both vertical and lateral directions, as shown in Fig. 1c.

The yield of nanosheets was measured by filtration of the dispersion liquid. The nanosheets in the dispersion liquid were collected by filtration using a membrane filter with 0.1 μm pore size. The yield of the nanosheets was around 30% after 1 h, 3 h, and 5 h of exfoliation (Fig. 8c). However, the yield was lowered after 24 h. The low yield is caused by the measurement method. Since the lateral size and thickness are decreased with an increase in the exfoliation time, the smaller nanosheets pass through the filter. Therefore, the measured yield was lower after 24 h.

The changes in the lateral size, thickness, and yield were studied for different exfoliation methods as the reference (the right panels in Fig. 8). In methods A–E, the dispersion liquid of the nanosheets was obtained without stirring at 60 °C. The nanosheets were collected by filtration after removal of the unexfoliated objects. The layered C_14_–TiO_2_ was maintained in toluene for 120 h without any mixing and stirring treatments (method A). The toluene dispersion liquid was maintained for 120 h and then shaken by hand for 10 s (method B). The toluene dispersion liquid was initially shaken for 10 s and then maintained for 120 h (method C). The dispersion liquid was maintained for 120 h and then placed in a sonic bath for 5 min (method D). The dispersion liquid was placed in a sonic bath for 5 min and then maintained for 120 h (method E). The reference exfoliation methods B–E showed no remarkable changes in the size, thickness, and yield compared with those obtained by the original method (Fig. 8). Although exfoliation method A showed the minimum value of thickness, the yield was the lowest in the present work. The results show that an external stimulus facilitates the exfoliation. However, the type of external stimulus is not a very important factor for determining the sizes of the nanosheets. The layered C_14_–TiO_2_ is gradually exfoliated into nanosheets through swelling of the hydrophobic interlayer space by toluene. In addition, the lateral size decreases with fracture of the thin layers in the dispersion media because of the instability originating from the thin structures in the dispersion media. Therefore, the thickness and lateral size are gradually decreased with an increase in the exfoliation time. These results imply that the chemical affinity between the guests and dispersion medium is a potential factor to control the size of the nanosheets derived from the soft layered composites.

This exfoliation method using soft layered composites can be applied to other inorganic layered compounds based on stacking *via* electrostatic interaction. In our previous studies, similar surface-modified nanosheets were obtained from layered manganese and tungsten oxides.^29,30^ Titanate nanosheets with different sizes were formed from different guest-medium combinations.^34,35^ In the present work, the layered composites of manganese oxide and C_14_–NH_2_ (C_14_–MnO_2_) were exfoliated into nanosheets in toluene. The layered C_14_–MnO_2_ was prepared by the method in our previous report.^29^ The exfoliation behavior was analyzed by DLS and AFM (Fig. S5 in the ESI†). Similar time-course changes of the nanosheet sizes including the lateral size and thickness were observed in the exfoliation of C_14_–MnO_2_ in toluene (Fig. 8).

### Potential for size control by the exfoliation method using soft layered composites

The present exfoliation method was compared to other methods and materials (Table S1 in the ESI†).^54–72^ Sonication in organic media is used for a variety of compounds, such as graphite, transition-metal dichalcogenides, boron nitride, black phosphor, tellurium, indium selenide, and nickel hydroxide.^54–65^ Typically, sonication in the liquid phase for 5–24 h induces exfoliation into nanosheets including few-layer and monolayer nanosheets. In addition, exfoliation of van der Waals stacks is achieved by shear stress using a food blender.^66,67^ A previous report showed that the lateral size and thickness of the nanosheets was not changed by the time of exfoliation.^66^ The size was changed by the concentration of the surfactant. Therefore, cascade centrifugation is generally used for selection of sizes.^8,9^ Exfoliation of layered compounds based on electrostatic interaction generally originates from the intercalation of bulky ions into the interlayer space.^15–19^ Layered double hydroxides consisting of cationic layers and anionic guest ions were exfoliated into nanosheets by sonication within 0.5–12 h in a polar organic medium, where the interlayer cation was changed to a larger anion, such as perchlorate.^68,69^ Exfoliation of layered transition-metal oxides was performed by intercalation of bulky cations, such as tetrabutylammonium, and subsequent osmotic swelling for 10 days or longer periods.^49,70,71^ However, it is not easy to control the lateral size and thickness of these nanosheets. In our method, the lateral size and thickness can be controlled by the exfoliation time within 1–120 h. The guest-medium combinations have the potential for synthesis of size-controlled nanosheets with tunable properties.

### Thickness-dependent changes of the properties

**Fig. 9:**
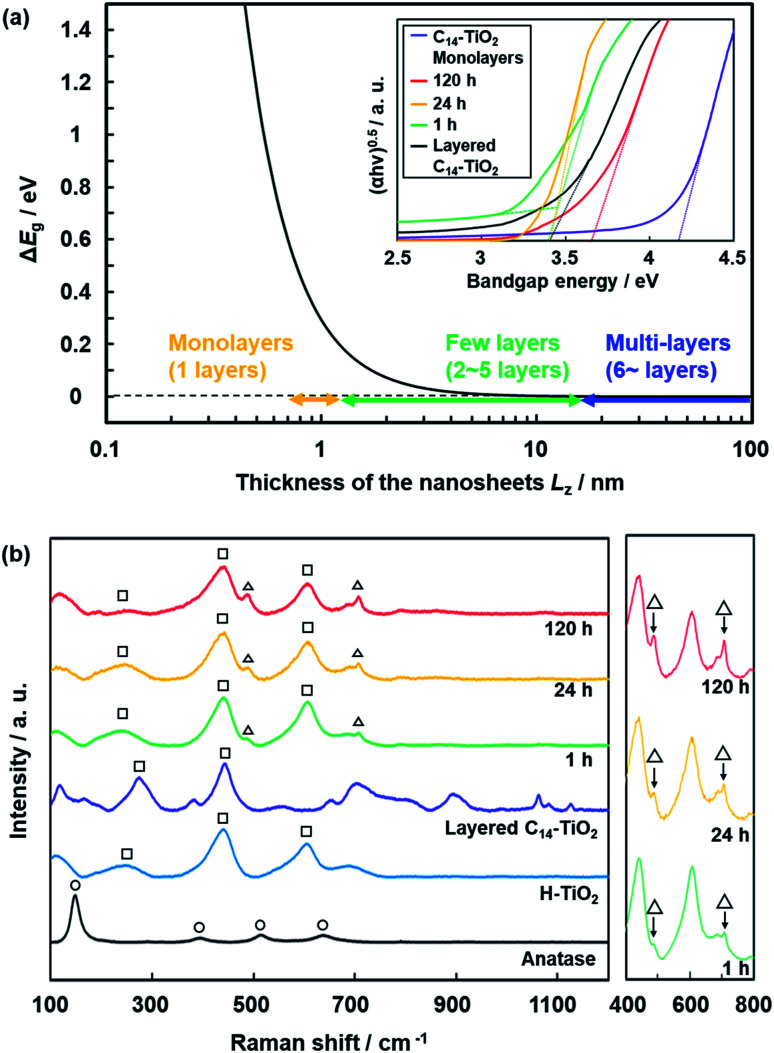
Thickness-dependent properties. (a) Relationship between the *L*_*z*_ and Δ*E*_g_ of the nanosheets calculated using eqn (1) and UV-Vis spectra of the layered C_14_–TiO_2_ nanosheets with exfoliation for 1, 24, and 120 h and C_14_–TiO_2_ monolayered nanosheets. (b) Raman spectra of the layered C_14_–TiO_2_ and H–TiO_2_, nanosheets with exfoliation for 1, 24, and 120 h, C_14_–TiO_2_ monolayered nanosheets, and anatase TiO_2_ as a reference. The peaks denoted by the triangles, squares, and circles indicate those corresponding to nanosheets, lepidocrocite-type titanium oxide, and anatase-type titanium oxide.

The thickness-dependent properties were studied by UV-Vis absorption and Raman spectroscopy (Fig. 9). The proportion of the monolayer and few-layer nanosheets was increased with an increase in the exfoliation time (Fig. 7). The bandgap energy (*E*_g_) was estimated from the Tauc plots of UV-Vis spectra (the inset of Fig. 9a). In our previous work, the bandgap energy (*E*_g_) of the layered C_14_–TiO_2_ and its monolayered surface-modified nanosheet was reported to be 3.42 eV and 4.06 eV, respectively.^29^ In the present work, the *E*_g_ of the resultant nanosheets was estimated to be 3.41 eV for the layered C_14_–TiO_2_, 3.40 eV after 1 h, 3.42 eV after 24 h, 3.67 eV after 120 h of, and 4.17 eV for the monolayered samples (the inset of Fig. 9a). The C_14_–TiO_2_ nanosheets obtained by exfoliation for 120 h showed a significant shift of the *E*_g_ (Δ*E*_g_), whereas such a shift was not observed for the other samples. The Δ*E*_g_ of semiconductor nanosheets is calculated using the following equation (eqn (1)),^29,71^ where *x* and *y* are the coordinates parallel to the nanosheet, *z* is the coordinate perpendicular to the sheet, *L*_*i*_ (*i* = *x*, *y* and *z*) is the lateral size and thickness, respectively, *μ*_*xy*_ and *μ*_*z*_ are the reduced effective masses of the electron–hole pair in the corresponding coordinates, and *h* is Planck's constant.1
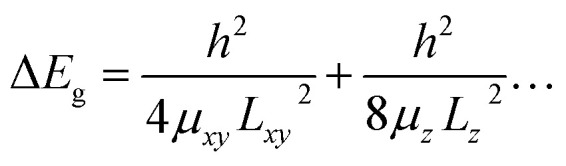
Here the thickness (*L*_*z*_)-dependent Δ*E*_g_ was calculated based on the assumption that *L*_*xy*_ = 10^3^ nm and *μ*_*xy*_ = *μ*_*z*_ = 1.63 *m*_e_, the same as that of bulk anatase titanium dioxide, where *m*_e_ is the mass of an electron (=9.11 × 10^−31^ kg) (Fig. 9a). Although previous works implied slight changes in the *μ*_*z*_ due to the thin nature and surface modification,^29,71^ these effects are ignored in the present calculation. The relationship between Δ*E*_g_ and *L*_*z*_ indicates that a significant increase in Δ*E*_g_ originating from the quantum-size effect can be observed below an *L*_*z*_ of around 5 nm (Fig. 9a). Since the C_14_–TiO_2_ nanosheet sample with exfoliation for 120 h has an average thickness of 4.8 ± 1.1 nm (Fig. 6l and 7), significant changes in the *E*_g_ (Δ*E*_g_ = 0.26 eV) were actually observed. On the other hand, such a significant shift is not found in the other samples with an average thickness larger than 7 nm (Fig. 6c, f, i and 7). The experimental and calculated results were consistent with each other.

Raman spectra showed new peaks for the C_14_–TiO_2_ nanosheet samples with a decrease in the average thickness (Fig. 9b). The layered H–TiO_2_ and C_14_–TiO_2_ showed peaks corresponding to lepidocrocite-type titanium oxide (the squares in Fig. 9b). The peak positions were different from those of anatase-type titanium oxide (the circles in Fig. 9b). The new peaks appeared around 490 cm^−1^ and 700 cm^−1^ for the C_14_–TiO_2_ nanosheet samples (the triangles in Fig. 9b). The intensity of these new peaks increased with a decrease in the average thickness (the right panel in Fig. 9b). The thickness-dependent peak shift of the Raman spectrum was reported for molybdenum disulfide nanosheets.^72^ These facts imply that the appearance of the new peaks in the present work originates from the monolayered and few-layered C_14_–TiO_2_ nanosheets. However, further studies, including calculation and theoretical analyses, are needed to understand the changes in the Raman spectra of the transition-metal-oxide nanosheets.

### Dispersion stability of the nanosheets

**Fig. 10:**
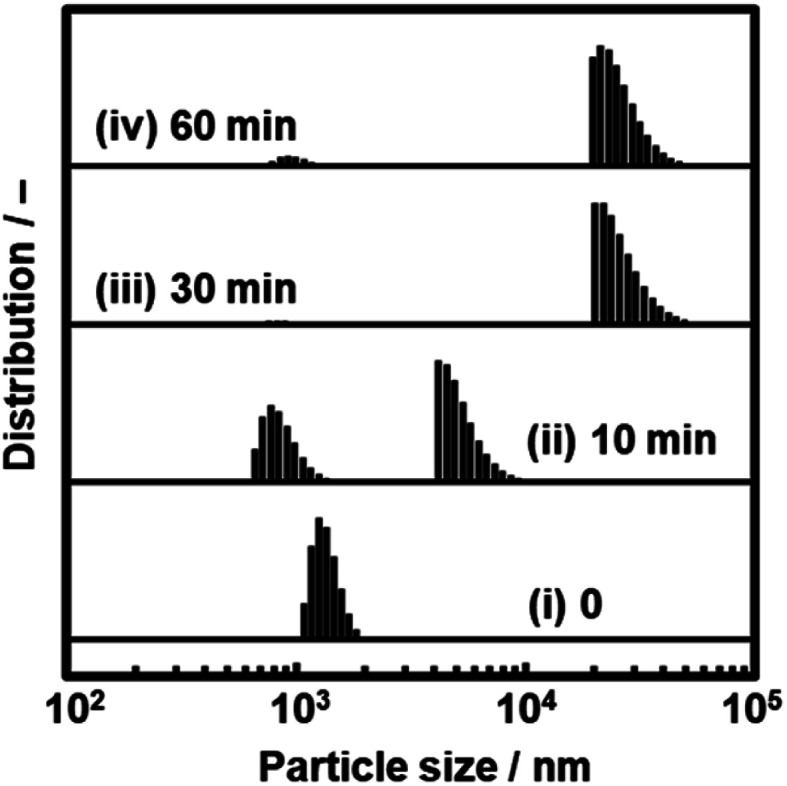
Time-course changes in the particle-size distribution of the dispersion liquid containing C_14_–TiO_2_ nanosheets (i) after exfoliation for 10 min (ii), 30 min (iii), and 60 min (iv).

Nanosheets are obtained in a colloidal liquid. Stability of the dispersion state is an important factor for their practical use. DLS analysis was used to study the time-course changes of the lateral size after exfoliation (Fig. 10). The C_14_–TiO_2_ nanosheets were obtained by exfoliation in toluene for 120 h at 60 °C under stirring. After removal of the unexfoliated objects, the DLS particle-size distribution showed a peak at 1.35 ± 0.18 μm (chart (i) in Fig. 10). The dispersion liquid was maintained in the DLS equipment to observe the changes of the particle-size distribution. After 10 min, an additional peak appeared in the region higher than around 5 μm (chart (ii) in Fig. 10). The main peak was shifted to the range of several tens of micrometers after 30 min and 60 min (charts (iii) and (iv) in Fig. 10). The aggregation of the resultant nanosheets gradually proceeds after exfoliation. All the nanosheets form aggregates after 30 min.

**Fig. 11:**
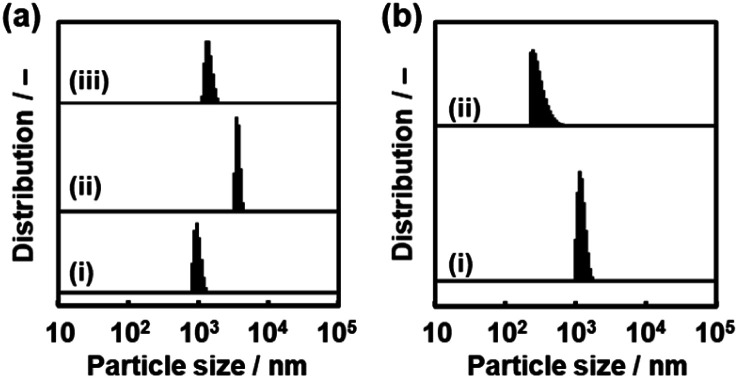
Redispersion behavior of the C_14_–TiO_2_ nanosheets in toluene measured by DLS. (a) Particle-size distribution of the as-prepared dispersion liquid (i), the dispersion liquid maintained for 1 h (ii), and the redispersion liquid with sonication for 5 min (iii). (b) Particle-size distribution of the as-prepared dispersion liquid (i) and redispersion liquid of the collected and dried powder with sonication for 5 min (ii).

We found that the aggregated nanosheets recovered to the original dispersion state with sonication for 5 min (Fig. 11). The toluene dispersion liquid containing nanosheets was prepared by exfoliation and then maintained for 1 h to induce aggregation. While the original dispersion liquid showed a particle-size distribution around 1 μm (chart (i) in Fig. 11a), the aggregation caused the peak to shift to a larger range (chart (ii) in Fig. 11a). The dispersion liquid with the aggregates was sonicated for 5 min at room temperature. The particle-size distribution recovered to the original state with the disappearance of the larger peak (chart (iii) in Fig. 11a).

Another important aspect is redispersibility of the nanosheets from the collected and dried state. The dispersed nanosheets were collected using a filter paper with a pore size of 0.1 μm. The dried nanosheets were then dispersed in toluene. Particle-size distribution similar to that of the original state was observed after sonication for 5 min (Fig. 11b). These results suggest that the original dispersion state recovers from the aggregated states by sonication. Since the rigid layered compound is converted to the soft composite with intercalation of the guests, the aggregates formed *via* van der Waals interaction can be easily detached in the dispersion media. According to these exfoliation and dispersion behaviors, the nanosheets derived from the layered composites can be manipulated as building blocks toward a wide range of applications. In our previous work, surface-modified nanosheets with a high specific surface area were used in catalytic, sensing, and photochemical applications.^29,31,32^ The size-controllable nanosheets have potential for tuning of these properties.

## Conclusions

The rigid layered compound was converted to the soft layered composite by intercalation of the guest. The layered composite of host titanate and guest amine was exfoliated into surface-modified nanosheets in an organic dispersion medium. Exfoliation of the soft layered composites was initiated by swelling with toluene introduced in the hydrophobic interlayer space. The lateral size and thickness decreased with an increase in the exfoliation time. The proportion of the monolayer and few-layer nanosheets increased with the exfoliation time, whereas that of the multilayer nanosheets decreased. The results suggest that the surface-functionalized nanosheets are generated from the soft layered composites by coupling exfoliation in the vertical direction and fracture in the lateral direction. Although aggregation of the nanosheets occurred in the dispersion liquid, the original dispersion state was recovered with sonication. These findings can be applied for controlled exfoliation of other rigid layered materials and manipulation of the resultant nanosheets. The layered composites can show different exfoliation behavior depending on the combination of the guest and medium. Although the structures and properties of the pristine layered compounds also have an effect on the exfoliation behavior, the guest-medium affinity is a control factor of the exfoliation process. The soft layered composites have the potential for controlling the exfoliation behavior.

## Conflicts of interest

There are no conflicts to declare.

## Supplementary Material

NA-002-D0NA00084A-s001
