# Supplementary material for: Formation processes, size changes, and properties of nanosheets derived from exfoliation of soft layered inorganic–organic composites
Source: Nanoscale Adv. 2020 Jan 30;2(3):1168–76. doi: 10.1039/d0na00084a (PMC9417460; doi:10.1039/d0na00084a)
Supplement: NA-002-D0NA00084A-s001 [file NA-002-D0NA00084A-s001.pdf]

Electronic Supplementary Material (ESI) for Nanoscale Advances.
This journal is © The Royal Society of Chemistry 2020

Electronic Supplementary Information (ESI)

Formation processes, size changes, and properties of the nanosheets derived from exfoliation of soft layered inorganic-organic composites

Ryosuke Mizuguchi, Hiroaki Imai, Yuya Oaki*

Department of Applied Chemistry, Faculty of Science and Technology, Keio University, 3-14-1 Hiyoshi, Kohoku-ku, Yokohama 223-8522, Japan

JST, PRESTO, 4-1-8 Honcho, Kawaguchi, Saitama 332-0012, Japan

E-mail: oakiyuya@applc.keio.ac.jp

Contents

Experimental methods P. S2

XRD patterns of the C_{14}-TiO_2 in the wet and dried states (Fig. S1) P. S4

TEM images, AFM images, and DLS charts (Fig. S2) P. S5

TG curves of the C_{14}-TiO_2 before and after the exfoliation (Fig. S3) P. S6

Schematic models of the C_{14}-TiO_2 monolayers and few-layers (Fig. S4) P. S7

Time-course exfoliation behavior of the C_{14}-MnO_2 (Fig. S5) P. S8

Comparison of the present exfoliation method with the other methods (Table S1) P. S9

Experimental methods

Synthesis of the layered C_{14}-TiO_2. All the reagents were used as purchased without purification. C_{14}-TiO_2 was synthesized from the precursor layered cesium titanate (Cs-TiO_2) through formation of protonated titanate (H-TiO_2), according to the previous works.[29] The starting Cs-TiO_2 was synthesized by solid-state reaction.[R1] The interlayer ion was exchanged from cesium ion to proton to obtain H-TiO_2 with through immersion in hydrochloric acid. The detailed concentration and condition were described in our previous work.[29] Then, the resultant H-TiO_2 as solid acid, typically 0.2 g, was dispersed in 5 cm^3 of an alkaline aqueous solution containing 16.4 mmol dm^{-3} tetradecylamine ($C_{14}H_{29}NH_2$, C_{14}-NH_2, TCI, 96.0 %) for 10 days at 25 °C under stirring. The molar ratio of the interlayer proton to C_{14}-NH_2 was adjusted to 1.0. The resultant C_{14}-TiO_2 was centrifuged, washed with purified water and ethanol, and then dried at room temperature.

Exfoliation of the layered C_{14}-TiO_2. The C_{14}-TiO_2 powder, typically 30 mg, was dispersed in 12 cm^3 of toluene at 60 °C under magnetic stirring around 300 rpm for certain period (1–120 h). The dispersion liquid was then filtered to remove the unexfoliated powder (pore size of the filter: 2.0 μm). The resultant supernatant was used as the colloidal liquid containing the C_{14}-TiO_2 nanosheets. Exfoliation of the C_{14}-MnO_2 was performed by the similar method according to our previous work.[29]

Characterization. The particle-size distribution of the C_{14}-TiO_2 nanosheets was measured by dynamic light scattering (DLS, Otsuka Electronics, ELSZ-2000ZS). The colloidal liquid was casted on a cleaned silicon substrate for atomic force microscopy (AFM, Shimadzu SPM-9700HT) observation and dropped on a collodion membrane for transmission electron microscopy (TEM, FEI Tecnai) observation operated at 120 kV. The C_{14}-TiO_2 nanosheet was collected by filtration of the dispersion liquid using a filter with pore size 0.1 μm to measure the yield. The crystal structure and interlayer distance were analyzed by X-ray diffraction (XRD, Bruker D8 Advance) with Cu-Kα radiation. The organic content of the layered C_{14}-TiO_2 and nanosheets was measured by thermogravimetry (TG) analysis (Seiko, TG-DTA 7000) in air atmosphere. The morphology of the layered C_{14}-TiO_2 was observed by field-emission scanning electron microscopy (FESEM, JEOL JSM-7600F) operated at 5.0 kV. The thermal properties of the layered composite were studied by DSC in nitrogen atmosphere (DSC, Shimadzu DSC-60). The UV-Vis spectra of the resultant C_{14}-TiO_2 nanosheets were measured in the dried

powder state by a spectrophotometer with an integrated sphere (JASCO V-670). Raman spectra were obtained in the dried powdered state on silicon substrate by excitation with laser 532 nm (Renishaw, inVia Raman Microscope).

Additional Reference

R1. T. Sasaki, Y. Komatsu and Y. Fujiki, *J. Chem. Soc. Chem. Commun.*, 1991, 817.

XRD patterns of the C_{14}-TiO_2 in the wet and dried states

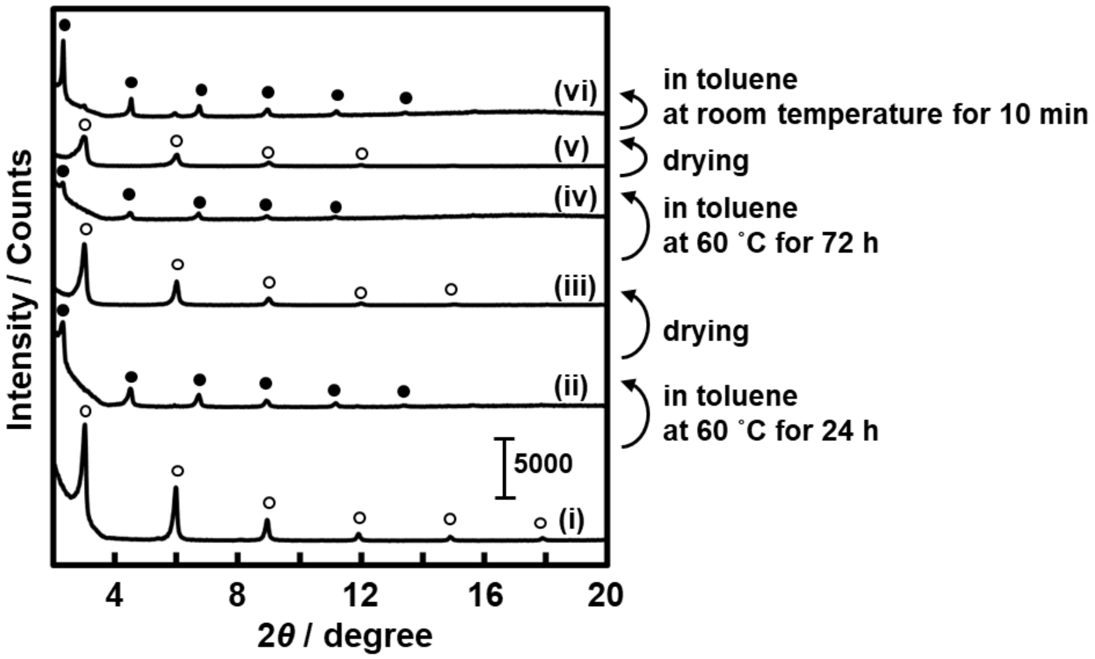

Fig. S1. Time-course XRD patterns of the original layered C_{14}-TiO_2 (i), wet state after immersion in toluene for 24 h at 60 °C (ii), subsequent dried state (iii), wet state after immersion in toluene for 72 h at 60 °C (iv), subsequent dried state (v), and wet state after immersion in toluene for 10 min at room temperature around 25 °C (vi).

A series of this dry-wet repetitive experiment were performed by the same sample in the order of (i) to (vi). The peak position was reversibly changed with immersion in toluene and drying. The dried and wet states showed the interlayer distance around d_0 = 2.9 nm (the open circles) and d_0 = 3.8 nm (the filed circles), respectively. The results indicate the swelling of the original layered C_{14}-TiO_2 with toluene. Moreover, the swelling state was achieved with immersion in toluene even for 10 min at room temperature (the pattern (vi) in Fig. S1).

TEM images, AFM images, and DLS charts

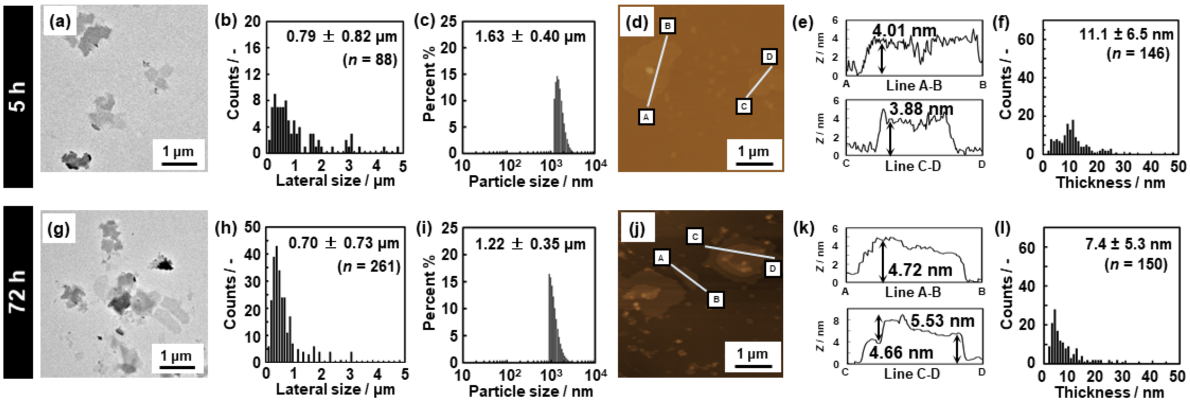

Fig. S2. Summary of the TEM images (a,g), histograms of the lateral size (b,h), DLS charts (c,i), AFM images (d,j), height profiles (e,k), and histograms of the height (f,l) of the nanosheets after exfoliation for 3 h (a–f) and 72 h (g–l).

Fig. 8 summarizes the data in Figs. 5 and 6 and Fig. S2.

TG curves of the C_{14}-TiO_2 before and after the exfoliation

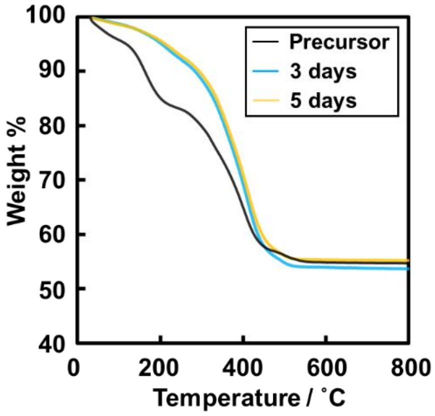

Fig. S3. TG curves of the original C_{14}-TiO_2 (black) and nanosheets after exfoliation in toluene at 60 °C for 3 (blue) and 5 (yellow) days.

The chemical formula of the C_{14}-TiO_2 was estimated to be $H_{0.7-x}(C_{14}\text{-}NH_2)_xTi_{1.825}O_4{\cdot}yH_2O$ according to our previous report.[R1,29,34] The x and y in the formula were calculated by TG analysis. These values were calculated to be $x = 0.49$, $y = 1.11$ for the original layered C_{14}-TiO_2 (i), $x = 0.55$, $y = 0.75$ for the nanosheets after exfoliation in toluene at 60 °C for 3 days (ii), $x = 0.53$, $y = 0.67$ for the nanosheets after exfoliation in toluene at 60 °C for 5 days (iii). The contents of the C_{14}-NH_2 were not changed even after the exfoliation. The results indicate that the surface C_{14}-NH_2 was not removed from the nanosheets in toluene.

Schematic models of the C_{14}-TiO_2 monolayers and few-layers

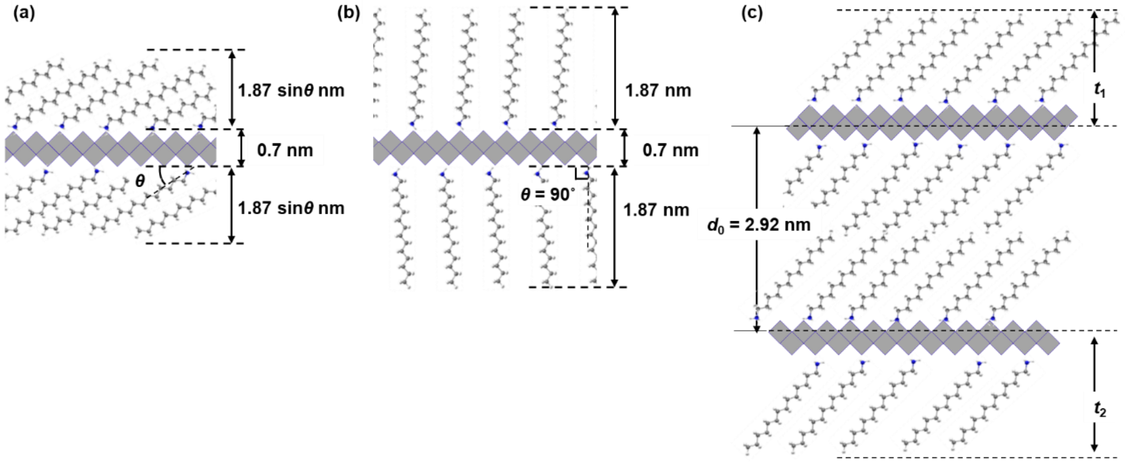

Fig. S4. Schematic models of the C_{14}-TiO_2 monolayers (a,b) and bilayer (c).

According to our previous reports, both the sides of the resultant nanosheets were modified by the alkylamine (Fig. S4a).[29] The thickness of the monolayered C_{14}-TiO_2 was estimated to be 1.5–2.0 nm by AFM analysis in our previous report (Fig. S4a).[29] The thickness of 1.5 nm corresponds to the tilted angle of the alkyl chain to the layer (θ) 12.3 °. The thickness of the monolayer (t_m / nm) is represented by (eq. S1) depending on θ (Fig. S4a), where the thickness of the bare titanate monolayer is assumed to be 0.7 nm according to the previous report and the molecular length of C_{14}-NH_2 is calculated to be 1.87 nm.[12,39]

$$t_m = 0.7 + 2 \times 1.87 \sin \theta \ldots \text{(eq. S1)}$$

If the alkyl chains are arranged perpendicular to the layer surface (θ = 90 °), the maximum thickness of the monolayer is estimated to be around 4.5 nm (Fig. S4b). Therefore, the thickness of the monolayer is defined as 1.50–4.50 nm in the present work. The thickness of the bilayer (t_b) structure is calculated to be 4.42–7.42 nm by (eq. S2) on the assumption that the interlayer space (d_0) is the same as that of the layered C_{14}-TiO_2 (d_0 = 2.92 nm) and the t_m is regarded as the sum of t_1 and t_2 in Fig. S4c.

$$t_b = t_m + d_0 \ldots \text{(eq. S2)}$$

In this manner, the thickness of the N-layered nanosheets (t_N) is calculated by (eq. S3).

$$T_N = t_m + (N - 1) \times d_0 \ldots \text{(eq. S3)}$$

Therefore, the thickness of the few-layer, namely N = 2–5, is defined as 4.50–16.5 nm. The nanosheets thicker than 16.5 nm, namely N > 5, is regarded as multi-layer.

Time-course exfoliation behavior of the C_{14}-MnO_2

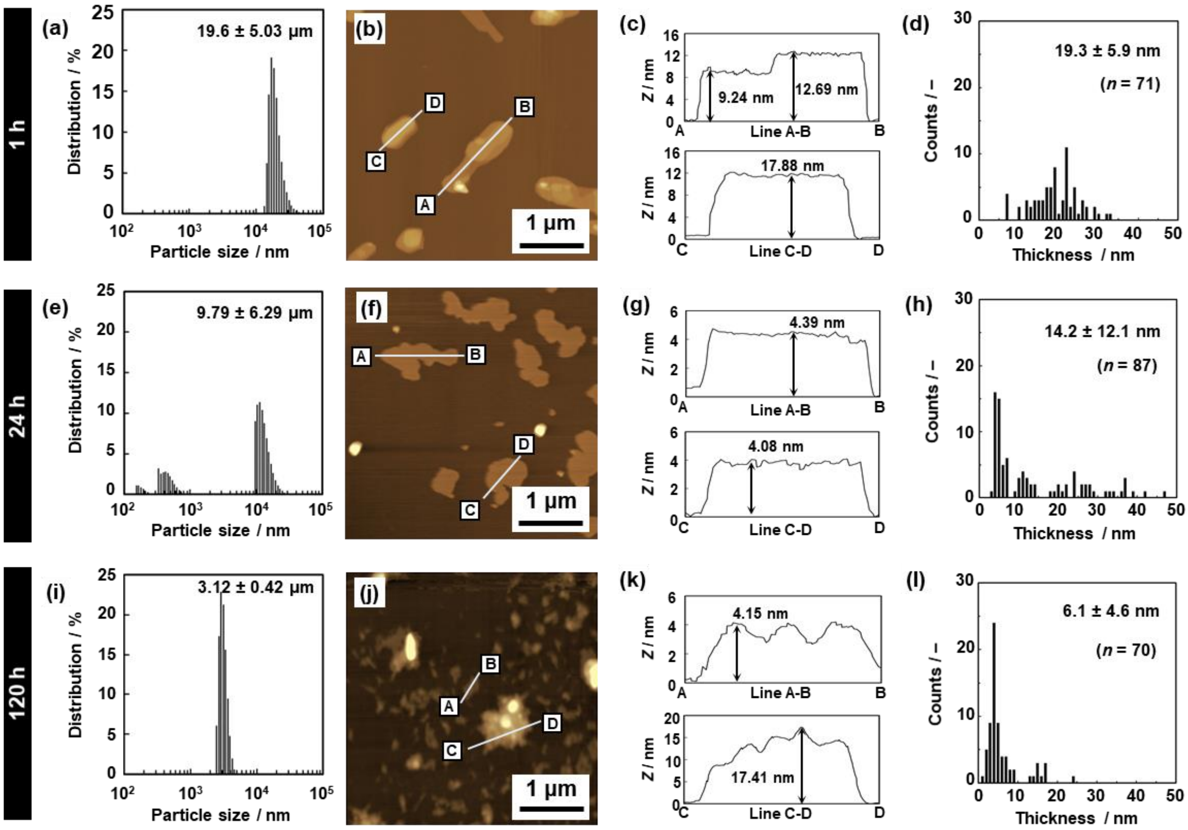

Fig. S5. Summary of the DLS charts (a,e,i), AFM images (b,f,g), height profiles (c,g,k), and histograms of the height (d,h,l) of the C_{14}-MnO_2 nanosheets after exfoliation for 1 h (a–d), 24 h (e–h), and 120 h (i–l).

The summarized data were shown in Fig. 8 in the main text.

Comparison of the present exfoliation method with the other methods

Table S1. List of typical exfoliation methods and materials in previous studies.[49,54–71]

No.	Materials	Time / h	Methods	ref
1	g-C_3N_4	0.5	Dispersion in aqueous solution	54
2	Graphite	0.5	Microwave exfoliation in an ionic liquid	55
3	[a]TMD	0.5	Redox reactions in liquid phase	56
4	[a]TMD	0.5	Sonication	57
5	BN	8	Sonication	58
6	Graphite	10	Sonication	59
7	Black phosphor	24	Sonication	60
8	[a]TMD	9	Sonication	61
9	Graphene	6	Sonication	62
10	Tellurium	5	Sonication	63
11	InSe	6	Sonication	64
12	$Ni(OH)_2$	6	Sonication	65
13	[a]TMD	1-10	Shear stress (blender)	66
14	Graphene	0.5-8	Shear stress (blender)	67
15	Mg-Al LDH	12	Sonication	68
16	Co-Fe LDH	0.5	Sonication	69
17	MnO_2	240	Dispersion in aqueous medium for osmotic swelling	70
18	TiO_2	240	Dispersion in aqueous medium for osmotic swelling	71
19	TiO_2	336-3024	Dispersion in aqueous medium for osmotic swelling	49
20	Layered Composite	1-120	Dispersion in organic media	Present work

[a]TMD: transition-metal dichalcogenide

In Table S1, the materials with numbers 1–11,13,14 and 12,15–20 correspond to the layered compounds consisting of van der Waals interaction and electrostatic interaction, respectively.
